# *RAD51B* (rs8017304 and rs2588809), *TRIB1* (rs6987702, rs4351379, and rs4351376), *COL8A1* (rs13095226), and *COL10A1* (rs1064583) Gene Variants with Predisposition to Age-Related Macular Degeneration

**DOI:** 10.1155/2019/5631083

**Published:** 2019-05-02

**Authors:** Alvita Vilkeviciute, Loresa Kriauciuniene, Romanas Chaleckis, Vytenis Pranas Deltuva, Rasa Liutkeviciene

**Affiliations:** ^1^Neuroscience Institute, Lithuanian University of Health Sciences, Medical Academy, Eiveniu 2, LT-50161 Kaunas, Lithuania; ^2^Department of Ophthalmology, Lithuanian University of Health Sciences, Medical Academy, Eiveniu 2, LT-50161 Kaunas, Lithuania; ^3^Gunma University Initiative for Advanced Research, Maebashi, Gunma 371-8512, Japan

## Abstract

**Background:**

Age-related macular degeneration (AMD) is a progressive neurodegenerative disease of a central part of the neural retina (macula) and a leading cause of blindness in elderly people. While it is known that the AMD is a multifactorial disease, genetic factors involved in lipid metabolism, inflammation, and neovascularization are currently being widely studied in genome-wide association studies (GWAS). The aim of our study was to evaluate the impact of new single nucleotide polymorphisms (SNPs) in *RAD51B*, *TRIB1*, *COL8A1*, and *COL10A1* genes on AMD development.

**Methods:**

Case-control study involved 254 patients diagnosed with early AMD, 244 patients with exudative AMD, and 942 control subjects. The genotyping of *RAD51B* (rs8017304 and rs2588809), *TRIB1* (rs6987702, rs4351379, and rs4351376), *COL8A1* (rs13095226), and *COL10A1* (rs1064583) was carried out using TaqMan assays by a real-time polymerase chain reaction (RT-PCR) method.

**Results:**

Statistically significant difference was found in genotype (TT, TC, and CC) distribution of *COL8A1* rs13095226 between exudative AMD and control groups (60.2%, 33.6%, and 6.1% vs. 64.9%, 32.3%, and 2.9%, respectively, *p* = 0.036). Also, comparing with TT+TC, rs13095226 CC genotype was associated with 3.5-fold increased odds of exudative AMD development (OR = 3.540; 95% CI: 1.415-8.856; *p* = 0.007).

**Conclusion:**

Our study revealed a strong association between a variant in *COL8A1* (rs13095226) and exudative AMD development.

## 1. Introduction

Age-related macular degeneration (AMD) is a progressive neurodegenerative disease of a central part of neural retina (macula) [1]. AMD is a leading cause of central vision loss, and while it is diagnosed in elderly people (in those aged 60 and over) [1], the first signs of the disease can occur in people aged 40 [2]. One of the main processes involved in AMD pathogenesis is called drusogenesis [3]. Drusen are described as small particles of lipid, protein, and collagen detachments accumulated between the retinal pigment epithelium (RPE) and the Bruch's membrane (BrM) in the retina [3]. RPE controls fluid transportation between the choriocapillaris and the retina including lipid transportation and metabolism as well as oxygen transportation [4]. The BrM is a five-layer extracellular matrix located between the RPE and the choroid. It regulates metabolic exchange between RPE cells and blood flow from the choroid through a semipermeable filtration barrier [5]. Any alteration in the structure of BrM might impact dysfunction of the RPE and the outer retina [6]. One of the most complicated biological processes which may affect BrM is ageing: age-related processes urge the accumulation of incompletely digested phospholipids [7], combining with oxidative stress results of lipid peroxidation [8] and lysosomal defects in photoreceptor outer segments [9]. Oxidative stress-induced lipid and protein accumulation leads to RPE injury or early RPE cell death with changes in BrM [10, 11]. Damaged RPE cells release huge amounts of different cytokines and chemokines which could result in chronic inflammation over time [12–14]. One of the cytokines is a vascular endothelial growth factor A (VEGF-A) which can also induce angiogenesis [15–17]. This process leads to a growth of new fragile and leaky vessels resulting in exudative hemorrhage and acute vision loss [18]. According to the Age-Related Eye Disease Study (AREDS), AMD can be classified into early, intermediate, and late stages [19]. Early AMD is described as the appearance of drusen and retinal pigment abnormalities; when at least one large druse, numerous medium-sized drusen, or geographic atrophy (GA) without extension to the center of the macula occurs, the intermediate stage of AMD is diagnosed; late AMD is divided into dry AMD with the GA of the RPE and neovascular AMD which is characterized by choroidal neovascularization with detachments of the RPE, hemorrhages, and/or scars [20]. While it is known that the AMD is a multifactorial disease, genetic factors involved in lipid metabolism, inflammation, and neovascularization are currently being widely studied in genome-wide association studies (GWAS). Products of a collagen gene family are composed of types I, II, III, and V of fibril-forming interstitial and type IV basement membrane collagens. Collagens keep the architecture and function of normal tissues as well as the structure and function of the extracellular matrix [21, 22]. Recent studies detected an intronic variant in *COL8A1* gene (rs13095226 T/C), suggesting an association with advanced AMD [23–27]. The *COL8A1* gene is located on human chromosome 3 and encodes one of the two alpha chains of type VIII collagen which is a central component of multiple basement membranes of corneal endothelial cells, including BrM, choroidal stroma, and endothelia of blood vessels, playing a role in the maintenance of vascular integrity and structure [28, 29]. BrM and new vessel formation play a key role in pathogenesis of AMD [30]. Another collagen gene family member is *COL10A1* gene, located on human chromosome 6, which encodes the alpha chain of type X collagen. This short-chain collagen is expressed by hypertrophic chondrocytes during endochondral ossification, and it was shown that the expression of *COL10A1* was significantly downregulated in patients with osteoarthritis [31], but its expression was higher in diverse solid tumors and correlated with tumor vasculature [32]. The new variant near *FRK/COL10A1* (rs1999930) was found to be associated with advanced AMD development [24] and suggested the *COL10A1* as a potential locus for future association studies. While the oxidative stress has been linked to the various types of DNA damage playing a significant role in ageing and age-related disorders [33, 34], few studies [35, 36] revealed a significant association between oxidative stress-linked DNA damage and AMD development. Recently, GWAS pinpointed new genetic variations in the *RAD51B* gene associated with AMD [25, 26, 37, 38]. RAD51B is involved in homologous recombinational repair of DNA double-strand breaks by promoting the activity of the central recombinase [39]. Absence of RAD51B protein is thought to disrupt the formation of the RAD51 nucleoprotein filament, which is the initial stage of homologous recombinational repair [40]. G-protein-coupled receptor-induced proteins, playing a role in the mitogen-activated protein kinases- (MAPK-) related signaling cascade [41, 42] which mediates cell proliferation, differentiation, and apoptosis [43] and can regulate lipid metabolism through this pathway [44], are encoded by tribbles pseudokinase 1 (*TRIB1*) gene located in human chromosome 8. It has also been suggested that the *TRIB1* expression is regulated by inflammatory stimulation [45]. There was found one SNP in *TRIB1* gene (rs6987702) to be associated with AMD in African American and Mexican American population [46], suggesting a significant role of *TRIB1* in AMD development.

Association studies of these SNPs, however, have not been conducted in both (early and exudative) AMD forms. Genetic differences between developments of early and exudative AMD still need to be researched and understood better which might lead to future studies with focus on certain molecular pathways involved in the pathogenesis of AMD. Also, genetic variations combined with other risk factors or molecular changes can benefit physicians in creating personalized genetic therapies and/or lifestyle programs for increased risk individuals.

Considering previously studied links between genetic variations and AMD, we chose three previously described SNPs in RAD51B (rs8017304), TRIB1 (rs6987702), and COL8A1 (rs13095226) and four new genetic loci in RAD51B (rs2588809), TRIB1 (rs4351376 and rs4351379), and COL10A1 (rs1064583) genes as potential biomarkers for early and exudative AMD. The aim of our study was to determine and evaluate their impact on the development of early and exudative AMD in the Lithuanian population.

## 2. Materials and Methods

The study was conducted in the Department of Ophthalmology, Hospital of Lithuanian University of Health Sciences and Laboratory of Ophthalmology, Neuroscience Institute, Lithuanian University of Health Sciences. Ethical approval was obtained from the Ethics Committee for Biomedical Research (number: BE-2-/13).

Ophthalmological evaluation, study group formation, DNA extraction and genotyping methods, and statistical analysis were described in detail in our previous studies [47, 48].

## 3. Results

### 3.1. Demographic Characteristics of the Study Groups

Our study involved 498 patients (254 patients with a diagnosis of early AMD and 244 patients with exudative AMD) and 942 healthy controls. The control group was formed of 942 subjects classified into different genders, which matched gender classification in the early and exudative AMD group structure; however, subjects of the control group were younger than AMD patients (*p* < 0.001) (Table 1).

### 3.2. Hardy-Weinberg Equilibrium

When genotyping results showed that G allele at *TRIB1* rs4351376 was observed in all study subjects (100%), it was not included in further analysis. Hardy-Weinberg equilibrium (HWE) analysis showed that the distribution of genotypes of rs6987702 and rs1064583 deviated from the HWE in the control group and those two SNPs were excluded from further statistical analysis as well (Table 2).

### 3.3. Analysis of Single Nucleotide Polymorphisms

Analysis of four SNPs (rs8017304, rs2588809, rs4351379, and rs13095226) in the early and exudative AMD and control groups showed a statistically significant difference only in genotype (TT, TC, and CC) distribution of *COL8A1* rs13095226, when compared to the exudative AMD and control groups (60.2%, 33.6%, and 6.1% vs. 64.9%, 32.3%, and 2.9%, respectively, *p* = 0.036) (Table 3).

Binomial logistic regression revealed that the rs13095226 CC genotype is associated with 3.5-fold increased odds of exudative AMD development under the recessive model (OR = 3.540; 95% CI: 1.415-8.856, *p* = 0.007) as well as under the codominant model (OR = 3.426; 95% CI: 1.355-8.667, *p* = 0.009) (Table 4). Results remained statistically significant even after applying Bonferroni correction (*p* < 0.05/4) (Table 4).

### 3.4. Haplotype Associations with AMD

In this section, we performed haplotype association analysis and evaluated the impact of haplotype constructed of two SNPs: rs8017304 and rs2588809 on early and exudative AMD development. Linkage disequilibrium (LD) analysis was assessed by both *D*′ and *r*^2^ measures. The *r*^2^ values were 0.055 and 0.063 in early AMD and exudative AMD analysis, respectively; for the haplotype block, |*D*′| values were 0.425 and 0.457 in early AMD and exudative AMD analysis, respectively.

Unfortunately, haplotype analysis did not reveal any significant associations with early or exudative AMD development after adjustment for age.

## 4. Discussion

Our study involved 498 AMD patients (254 patients with the diagnosis of early AMD and 244 patients with exudative AMD) and 942 healthy control subjects. Since we chose 7 SNPs, 5 of those were newly selected as risk factors for AMD development, *RAD51B* (rs2588809), *TRIB1* (rs6987702, rs4351376, and rs4351379), and *COL10A1* (rs1064583), and 2 were already associated with AMD development, *RAD51B* (rs8017304) and *COL8A1* (rs13095226). Unfortunately, our study revealed only one SNP (rs13095226) which was associated with 3.5-fold increased odds for exudative AMD development in the population of Lithuania (*p* = 0.007).

One of the SNPs near the *TRIB1* locus called rs17321515 was found affecting lipoprotein metabolism. It was strongly associated with triglycerides (TG), low-density lipoprotein cholesterol (LDL-C), and high-density lipoprotein cholesterol (HDL-C) and even with increased risk of coronary heart disease (CHD), but results remain controversial among the studies [49–51]. Another SNP (rs2954021) located in *TRIB1* gene was associated with increasing plasma TG levels and coronary artery disease (CAD) risk [52, 53] as well as the other three variants: rs2001945, rs2001845, and rs2001844 [54]. Furthermore, rs2001844 was associated with increased plasma TG and reduced HDL-C [54]. Several association studies reported a significant association between HDL-C and AMD development as well [55–59]; unfortunately, others did not reveal any significant associations between HDL-C and AMD development [60–66]. On the other hand, while the association of HDL-C and AMD is controversial, variant in lipid trait-associated gene *TRIB1* rs6987702 showed a significant association with AMD development, but those results did not survive strict corrections for multiple testing [46]. In our study, none of the SNPs in *TRIB1* reached a statistically significant association with early or exudative AMD development.

Studies of variants in *RAD51B* revealed statistically significant associations of SNPs in *RAD51B* with breast cancer occurrence [67–70]. Also, *RAD51B* rs911263 was associated with rheumatoid arthritis (RA) [71, 72]; two SNPs (rs11158728 and rs927220) were associated with nasopharyngeal carcinoma development [73], and one (rs34094401) was associated with Parkinson's disease [74]. Also, literature review explains that risk alleles at *RAD51B* rs8017304, rs13081855 near *COL8A1/FILIP1L* locus, and rs3812111 in *COL10A1* are associated with a greater risk for advanced AMD development [25]. Other three GWAS proved *RAD51B* gene association with AMD as well [26, 37, 38]. Seddon et al. conducted a 12-year follow-up study of 2765 individuals and revealed that *RAD51B* (HR: 0.8; 95% CI: 0.60-0.97, *p* = 0.03) was significantly related to AMD progression [26]. In another study, Seddon and coauthors [38] analyzed the progression of AMD and found 834 from 2951 subjects, who progressed from no AMD, early AMD, or intermediate AMD to an advanced AMD form, and also confirmed that *RAD51B* was associated with AMD progression. Fritsche et al. [25] also proved a strong *RAD51B* association (*p* < 5 × 10^−8^) with advanced AMD in Europeans and Asians. Chu et al. analyzed *RAD51B* influence on AMD using two cohorts from Caucasian and Han Chinese populations, as well. Scientists identified two new SNPs in *RAD51B* (rs17105278 and rs4902566) and confirmed the association between rs8017304 and AMD development in Caucasians [37], suggesting a significant role of *RAD51B* associated with DNA damage/DNA repair mechanism in AMD pathogenesis.

Recent studies indicated that variants in *COL8A1* gene were associated with AMD development [24–26, 38]. One of the GWAS studies was performed on 979 patients with advanced AMD and 1709 control subjects and revealed a significant association between rs13095226 and AMD (*p* = 2.5*e* − 06) in the European population [23]. Also, in this study, authors found that the frequencies of minor allele C at rs13095226 in patients with polypoidal choroidal vasculopathy (PCV), neovascular AMD (nAMD), and controls were similar to the frequencies in Americans, while differences were found between Americans and Asians [23]. Meta-analysis of advanced AMD confirmed previously found associations to advanced AMD [24], while in contrast, Yang et al.'s study including 300 cases with nAMD, 300 cases with PCV, and 300 control subjects did not show the association of *COL8A1* rs13095226 with nAMD or PCV, suggesting that this variant may not be a risk factor for nAMD or PCV in Chinese subjects [75]. Also, another variant of the same gene, polymorphism of *COL8A1* rs13081855, was linked to exudative AMD development [27]. As in non-AMD patients, one of the common variants in *COL8A1* gene (rs669676) was found to be associated with myopic choroidal neovascularization (OR: 1.88; 95% CI: 1.18-2.98, *p* = 0.0076) but did not survive multiple testing [74]. Our study of the Lithuanian population discovered a strong association between a variant in *COL8A1* (rs13095226) and exudative AMD development; genotype rs13095226 CC was determined to be associated with 3.5-fold increased odds of exudative AMD development.

We also found a study, which showed the contribution of the extracellular collagen matrix (FRK/COL10A1) pathways to the development of advanced AMD. A coding variant rs3812111 in *COL10A1*, a collagen protein, could be involved in maintaining the structure and function of the extracellular matrix [24]. Ferrington et al. analyzed that the *COL10A1* rs3812111 A/T (nonrisk allele/risk allele) ratio was 0.69/0.65 in controls/age-related macular degeneration [76]. To our knowledge, there are no studies analyzing *COL10A1* (rs1064583) polymorphism in patients with AMD.

The following limitations of this study have to be declared:

- Patients with atrophic AMD have not been analyzed
- Sample size of AMD and healthy controls was relatively small
- Possibility of other risk factors was not included in the study

A thorough medical examination of the study objects can be acknowledged as one of the main strengths of our study. All patients were examined for chronic infectious and noninfectious diseases by a general practitioner. It is also important to highlight that RAD51B (rs8017304 and rs2588809), TRIB1 (rs6987702, rs4351379, and rs4351376), COL8A1 (rs13095226), and COL10A1 (rs1064583) gene variants have never been studied in the Baltic states, namely, in the Lithuanian population, and our study was the first of its type.

## 5. Conclusion

Our study revealed a strong association between variant in *COL8A1* (rs13095226) and exudative AMD development.

## Figures and Tables

**Table 1:** Demographic characteristics.

Characteristic	Group			*p* value
	Early AMD (*n* = 254)	Exudative AMD (*n* = 244)	Control (*n* = 942)	
Gender				
Male, *n* (%)	83 (32.7)	87 (35.7)	350 (37.2)	0.188^∗^
Female, *n* (%)	171 (67.3)	157 (64.3)	592 (62.8)	0.665^∗∗^

Age (years), median (IQR)	73 (12)	76 (11)	53 (0)	<0.001^∗^ <0.001^∗∗^

^∗^Early AMD group vs. the control group. ^∗∗^Exudative AMD group vs. the control group.

**Table 2:** Analysis of Hardy-Weinberg equilibrium in control group subjects.

SNP	Allele frequencies		Genotype distribution in the control group	*p* value
*RAD51B* rs8017304	G (0.31)	A (0.69)	90/401/451	0.950
*RAD51B* rs2588809	T (0.12)	C (0.88)	17/185/740	0.175
*TRIB1* rs6987702	C (0.27)	T (0.73)	83/343/516	**0.019**
*TRIB1* rs4351379	C (0.07)	G (0.94)	7/109/826	0.111
*COL8A1* rs13095226	C (0.19)	T (0.81)	27/304/611	0.138
*COL10A1* rs1064583	G (0.31)	A (0.69)	52/482/408	**<0.001**

**Table 3:** *RAD51B* (rs8017304 and rs2588809), *TRIB1* (rs4351379), and *COL8A1* (rs13095226) SNPs in patients with early and exudative AMD and control groups.

Gene/marker	Genotype/allele	Early AMD, *n* (%)	Control group, *n* (%)	*p* value	Exudative AMD, *n* (%)	Control group, *n* (%)	*p* value
*RAD51B* rs8017304	AA	125 (49.2)	451 (47.9)		117 (48.0)	451 (47.9)	
	AG	102 (40.2)	401 (42.6)	0.745	110 (45.1)	401 (42.6)	0.423
	GG	27 (10.6)	90 (9.6)		17 (7.0)	90 (9.6)	
	A	352 (69.3)	1303 (69.2)	0.955	544 (70.5)	1303 (69.2)	
	G	156 (30.7)	581 (30.8)		144 (29.5)	581 (30.8)	0.570

*RAD51B* rs2588809	CC	196 (77.2)	740 (78.6)		188 (77.0)	740 (78.6)	
	CT	50 (19.7)	185 (19.6)	0.410	53 (21.7)	185 (19.6)	0.652
	TT	8 (3.1)	17 (1.8)		3 (1.2)	17 (1.8)	
	C	442 (87.0)	1665 (88.4)	0.398	429 (87.9)	1665 (88.4)	
	T	66 (13.0)	219 (11.6)		59 (12.1)	219 (11.6)	0.775

*TRIB1* rs4351379	GG	214 (84.3)	826 (87.7)		217 (88.9)	826 (87.7)	
	GC	39 (15.4)	109 (11.6)	0.229	26 (10.7)	109 (11.6)	0.780
	CC	1 (0.4)	7 (0.7)		1 (0.4)	7 (0.7)	
	G	467 (91.9)	1761 (93.5)	0.222	460 (94.3)	1761 (93.5)	
	C	41 (8.1)	123 (6.5)		28 (5.7)	123 (6.5)	0.524

*COL8A1* rs13095226	TT	166 (65.4)	611 (64.9)		147 (60.2)	611 (64.9)	
	TC	74 (29.1)	304 (32.3)	0.095	82 (33.6)	304 (32.3)	**0.036**
	CC	14 (5.5)	27 (2.9)		15 (6.1)	27 (2.9)	
	T	406 (79.9)	1526 (81.0)	0.585	376 (77.0)	1526 (81.0)	
	C	102 (20.1)	358 (19.0)		112 (23.0)	358 (19.0)	0.051

AMD: age-related macular degeneration. *p* value < 0.05 indicated in bold is statistically significant.

**Table 4:** Binomial logistic regression analysis of the *COL8A1* (rs13095226) in patients with exudative AMD and controls.

Exudative AMD			AIC value
SNP	Model/genotype	OR; 95% CI; p^∗^	
	*Codominant*		
rs13095226	TC vs. TT CC vs. TT	0.906; 0.589-1.394; 0.653 3.426; 1.355-8.667; **0.009**	640.511
	*Recessive*		
rs13095226	CC vs. TT+TC	3.540; 1.415-8.856; **0.007**	638.713

^∗^ *p* was adjusted for age in logistic regression models. OR: odds ratio; 95% CI: 95% confidence interval; AIC: akaike information criterion.

## Data Availability

The genotyping data used to support the findings of this study are available from the corresponding author upon request.

## References

[1] Wong W. L., Su X., Li X. (2014). Global prevalence of age-related macular degeneration and disease burden projection for 2020 and 2040: a systematic review and meta-analysis. *The Lancet Global Health*.

[2] Klein R., Klein B. E. K., Jensen S. C., Meuer S. M. (1997). The five-year incidence and progression of age-related maculopathy: the Beaver Dam Eye Study. *Ophthalmology*.

[3] Ferris F. L., Davis M. D., Clemons T. E. (2005). A simplified severity scale for age-related macular degeneration: AREDS Report No. 18. *Archives of Ophthalmology*.

[4] Miller J. W. (2016). Beyond VEGF—the Weisenfeld lecture. *Investigative Ophthalmology & Visual Science*.

[5] Hogan M. J. (1972). Role of the retinal pigment epithelium in macular disease. *Transactions - American Academy of Ophthalmology and Otolaryngology*.

[6] Hewitt A. T., Newsome D. A. (1985). Altered synthesis of Bruch’s membrane proteoglycans associated with dominant retinitis pigmentosa. *Current Eye Research*.

[7] Finnemann S. C., Leung L. W., Rodriguez-Boulan E. (2002). The lipofuscin component A2E selectively inhibits phagolysosomal degradation of photoreceptor phospholipid by the retinal pigment epithelium. *Proceedings of the National Academy of Sciences of the United States of America*.

[8] Suzuki M., Kamei M., Itabe H. (2007). Oxidized phospholipids in the macula increase with age and in eyes with age-related macular degeneration. *Molecular Vision*.

[9] Ershov A. V., Bazan N. G. (2000). Photoreceptor phagocytosis selectively activates PPAR*γ* expression in retinal pigment epithelial cells. *Journal of Neuroscience Research*.

[10] Del Priore L. V., Kuo Y. H., Tezel T. H. (2002). Age-related changes in human RPE cell density and apoptosis proportion in situ. *Investigative Ophthalmology and Visual Science*.

[11] Wang A. L., Lukas T. J., Yuan M., Du N., Tso M. P., Neufeld A. H. (2009). Autophagy, exosomes and drusen formation in age-related macular degeneration. *Autophagy*.

[12] Bhutto I., Lutty G. (2012). Understanding age-related macular degeneration (AMD): relationships between the photoreceptor/retinal pigment epithelium/Bruch’s membrane/choriocapillaris complex. *Molecular Aspects of Medicine*.

[13] Parmeggiani F., Romano M. R., Costagliola C. (2012). Mechanism of inflammation in age-related macular degeneration. *Mediators of Inflammation*.

[14] Xu H., Chen M., Forrester J. V. (2009). Para-inflammation in the aging retina. *Progress in Retinal and Eye Research*.

[15] Lin T., Walker G. B., Kurji K. (2013). Parainflammation associated with advanced glycation endproduct stimulation of RPE in vitro: implications for age-related degenerative diseases of the eye. *Cytokine*.

[16] Bhutto I. A., McLeod D. S., Hasegawa T. (2006). Pigment epithelium-derived factor (PEDF) and vascular endothelial growth factor (VEGF) in aged human choroid and eyes with age-related macular degeneration. *Experimental Eye Research*.

[17] Das A., McGuire P. G. (2003). Retinal and choroidal angiogenesis: pathophysiology and strategies for inhibition. *Progress in Retinal and Eye Research*.

[18] Coleman H. R., Chan C. C., Ferris F. L., Chew E. Y. (2008). Age-related macular degeneration. *The Lancet*.

[19] The Age-Related Eye Disease Study Research Group (2001). The Age-Related Eye Disease Study system for classifying age-related macular degeneration from stereoscopic color fundus photographs: the Age-Related Eye Disease Study Report Number 6. *American Journal of Ophthalmology*.

[20] Ryan S. J. (2006). *Retina*.

[21] McCarthy J. B., Vachhani B., Iida J. (1996). Cell adhesion to collagenous matrices. *Biopolymers*.

[22] Engel J. (1997). Versatile collagens in invertebrates. *Science*.

[23] Neale B. M., Fagerness J., Reynolds R. (2010). Genome-wide association study of advanced age-related macular degeneration identifies a role of the hepatic lipase gene (LIPC). *Proceedings of the National Academy of Sciences of the United States of America*.

[24] Yu Y., Bhangale T. R., Fagerness J. (2011). Common variants near FRK/COL10A1 and VEGFA are associated with advanced age-related macular degeneration. *Human Molecular Genetics*.

[25] The AMD Gene Consortium (2013). Seven new loci associated with age-related macular degeneration. *Nature Genetics*.

[26] Seddon J. M., Reynolds R., Yu Y., Rosner B. (2014). Three new genetic loci (R1210C in CFH, variants in COL8A1 and RAD51B) are independently related to progression to advanced macular degeneration. *PLoS One*.

[27] Cascella R., Strafella C., Longo G. (2018). Uncovering genetic and non-genetic biomarkers specific for exudative age-related macular degeneration: significant association of twelve variants. *Oncotarget*.

[28] Tamura Y., Konomi H., Sawada H., Takashima S., Nakajima A. (1991). Tissue distribution of type VIII collagen in human adult and fetal eyes. *Investigative Ophthalmology & Visual Science*.

[29] Xu R., Yao Z. Y., Xin L., Zhang Q., Li T. P., Gan R. B. (2001). NC1 domain of human type VIII collagen (*α* 1) inhibits bovine aortic endothelial cell proliferation and causes cell apoptosis. *Biochemical and Biophysical Research Communications*.

[30] de Jong P. T. V. M. (2006). Age-related macular degeneration. *The New England Journal of Medicine*.

[31] Lamas J. R., Rodriguez-Rodriguez L., Vigo A. G. (2010). Large-scale gene expression in bone marrow mesenchymal stem cells: a putative role for COL10A1 in osteoarthritis. *Annals of the Rheumatic Diseases*.

[32] Chapman K. B., Prendes M. J., Sternberg H. (2012). COL10A1 expression is elevated in diverse solid tumor types and is associated with tumor vasculature. *Future Oncology*.

[33] Singh D. K., Ahn B., Bohr V. A. (2009). Roles of RECQ helicases in recombination based DNA repair, genomic stability and aging. *Biogerontology*.

[34] Wilson D. M., Bohr V. A. (2007). The mechanics of base excision repair, and its relationship to aging and disease. *DNA Repair*.

[35] Szaflik J. P., Janik-Papis K., Synowiec E. (2009). DNA damage and repair in age-related macular degeneration. *Mutation Research*.

[36] Wozniak K., Szaflik J. P., Zaras M. (2009). DNA damage/repair and polymorphism of the *hOGG1* gene in lymphocytes of AMD patients. *Journal of Biomedicine and Biotechnology*.

[37] Chu X. K., Meyerle C. B., Liang X., Chew E. Y., Chan C.-C., Tuo J. (2014). In-depth analyses unveil the association and possible functional involvement of novel *RAD51B* polymorphisms in age-related macular degeneration. *Age*.

[38] Seddon J. M., Silver R. E., Kwong M., Rosner B. (2015). Risk prediction for progression of macular degeneration: 10 common and rare genetic variants, demographic, environmental, and macular covariates. *Investigative Ophthalmology & Visual Science*.

[39] Suwaki N., Klare K., Tarsounas M. (2011). RAD51 paralogs: roles in DNA damage signalling, recombinational repair and tumorigenesis. *Seminars in Cell & Developmental Biology*.

[40] Takata M., Sasaki M. S., Sonoda E. (2000). The Rad51 paralog Rad51B promotes homologous recombinational repair. *Molecular and Cellular Biology*.

[41] Kiss-Toth E., Bagstaff S. M., Sung H. Y. (2004). Human tribbles, a protein family controlling mitogen-activated protein kinase cascades. *The Journal of Biological Chemistry*.

[42] Hegedus Z., Czibula A., Kiss-Toth E. (2007). Tribbles: a family of kinase-like proteins with potent signalling regulatory function. *Cellular Signalling*.

[43] Zareen N., Biswas S. C., Greene L. A. (2013). A feed-forward loop involving Trib3, Akt and FoxO mediates death of NGF-deprived neurons. *Cell Death and Differentiation*.

[44] Burkhardt R., Toh S. A., Lagor W. R. (2010). Trib1 is a lipid- and myocardial infarction-associated gene that regulates hepatic lipogenesis and VLDL production in mice. *The Journal of Clinical Investigation*.

[45] Sung H. Y., Francis S. E., Crossman D. C., Kiss-Toth E. (2006). Regulation of expression and signalling modulator function of mammalian tribbles is cell-type specific. *Immunology Letters*.

[46] Restrepo N. A., Spencer K. L., Goodloe R. (2014). Genetic determinants of age-related macular degeneration in diverse populations from the PAGE study. *Investigative Ophthalmology & Visual Science*.

[47] Liutkeviciene R., Vilkeviciute A., Kriauciuniene L., Deltuva V. P. (2019). SIRT1 rs12778366, FGFR2 rs2981582, STAT3 rs744166, LIPC rs10468017, rs493258 and LPL rs12678919 genotypes and haplotype evaluation in patients with age-related macular degeneration. *Gene*.

[48] Liutkeviciene R., Vilkeviciute A., Streleckiene G., Kriauciuniene L., Chaleckis R., Deltuva V. P. (2017). Associations of cholesteryl ester transfer protein (CETP) gene variants with predisposition to age-related macular degeneration. *Gene*.

[49] Aung L. H. H., Yin R.-X., Wu D.-F. (2011). Association of the TRIB1 tribbles homolog 1 gene rs17321515 A>G polymorphism and serum lipid levels in the Mulao and Han populations. *Lipids in Health and Disease*.

[50] Wang L., Jing J., Fu Q. (2015). Association study of genetic variants at newly identified lipid gene TRIB1 with coronary heart disease in Chinese Han population. *Lipids in Health and Disease*.

[51] Hegele R. A., Ban M. R., Hsueh N. (2009). A polygenic basis for four classical Fredrickson hyperlipoproteinemia phenotypes that are characterized by hypertriglyceridemia. *Human Molecular Genetics*.

[52] Varga T. V., Sonestedt E., Shungin D. (2014). Genetic determinants of long-term changes in blood lipid concentrations: 10-year follow-up of the GLACIER study. *PLoS Genetics*.

[53] Waterworth D. M., Ricketts S. L., Song K. (2010). Genetic variants influencing circulating lipid levels and risk of coronary artery disease. *Arteriosclerosis, Thrombosis, and Vascular Biology*.

[54] Douvris A., Soubeyrand S., Naing T. (2014). Functional analysis of the TRIB*1* associated locus linked to plasma triglycerides and coronary artery disease. *Journal of the American Heart Association*.

[55] Nowak M., Swietochowska E., Marek B. (2005). Changes in lipid metabolism in women with age-related macular degeneration. *Clinical and Experimental Medicine*.

[56] Reynolds R., Rosner B., Seddon J. M. (2010). Serum lipid biomarkers and hepatic lipase gene associations with age-related macular degeneration. *Ophthalmology*.

[57] Hogg R. E., Woodside J. V., Gilchrist S. E. C. M. (2008). Cardiovascular disease and hypertension are strong risk factors for choroidal neovascularization. *Ophthalmology*.

[58] Mitchell P., Wang J. J., Foran S., Smith W. (2002). Five-year incidence of age-related maculopathy lesions: the Blue Mountains Eye Study. *Ophthalmology*.

[59] Ulas F., Balbaba M., Ozmen S., Celebi S., Dogan U. (2013). Association of dehydroepiandrosterone sulfate, serum lipids, C-reactive protein and body mass index with age-related macular degeneration. *International Ophthalmology*.

[60] Klein R., Cruickshanks K. J., Nash S. D. (2010). The prevalence of age-related macular degeneration and associated risk factors. *Archives of Ophthalmology*.

[61] Cackett P., Wong T. Y., Aung T. (2008). Smoking, cardiovascular risk factors, and age-related macular degeneration in Asians: the Singapore Malay Eye Study. *American Journal of Ophthalmology*.

[62] Chakravarthy U., Wong T. Y., Fletcher A. (2010). Clinical risk factors for age-related macular degeneration: a systematic review and meta-analysis. *BMC Ophthalmology*.

[63] Tomany S. C., Wang J. J., van Leeuwen R. (2004). Risk factors for incident age-related macular degeneration: pooled findings from 3 continents. *Ophthalmology*.

[64] Delcourt C., Michel F., Colvez A., Lacroux A., Delage M., Vernet M. H. (2001). Associations of cardiovascular disease and its risk factors with age-related macular degeneration: the POLA study. *Ophthalmic Epidemiology*.

[65] Hyman L., Schachat A. P., He Q., Leske M. C. (2000). Hypertension, cardiovascular disease, and age-related macular degeneration. Age-Related Macular Degeneration Risk Factors Study Group. *Archives of Ophthalmology*.

[66] Buch H. (2005). 14-year incidence of age-related maculopathy and cause-specific prevalence of visual impairment and blindness in a Caucasian population: the Copenhagen City Eye Study. *Acta Ophthalmologica Scandinavica*.

[67] Thomas G., Jacobs K. B., Kraft P. (2009). A multi-stage genome-wide association in breast cancer identifies two novel risk alleles at 1p11.2 and 14q24.1 (RAD51L1). *Nature Genetics*.

[68] Figueroa J. D., Garcia-Closas M., Humphreys M. (2011). Associations of common variants at 1p11.2 and 14q24.1 (RAD51L1) with breast cancer risk and heterogeneity by tumor subtype: findings from the Breast Cancer Association Consortium. *Human Molecular Genetics*.

[69] Shu X. O., Long J., Lu W. (2012). Novel genetic markers of breast cancer survival identified by a genome-wide association study. *Cancer Research*.

[70] Orr N., Lemnrau A., Cooke R. (2012). Genome-wide association study identifies a novel variant in RAD51B associated with male breast cancer risk. *Nature Genetics*.

[71] Zhi L., Yao S., Ma W. (2017). Polymorphisms of RAD51B are associated with rheumatoid arthritis and erosion in rheumatoid arthritis patients. *Scientific Reports*.

[72] McAllister K., Yarwood A., Bowes J. (2013). Brief report: identification of BACH2 and RAD51B as rheumatoid arthritis susceptibility loci in a meta-analysis of genome-wide data. *Arthritis and Rheumatism*.

[73] Qin H.-D., Shugart Y. Y., Bei J.-X. (2011). Comprehensive pathway-based association study of DNA repair gene variants and the risk of nasopharyngeal carcinoma. *Cancer Research*.

[74] Sandor C., Honti F., Haerty W. (2017). Whole-exome sequencing of 228 patients with sporadic Parkinson’s disease. *Scientific Reports*.

[75] Yu Y., Huang L., Wang B., Zhang C., Bai Y., Li X. (2015). COL8A1 rs13095226 polymorphism shows no association with neovascular age-related macular degeneration or polypoidal choroidal vasculopathy in Chinese subjects. *International Journal of Clinical and Experimental Pathology*.

[76] Ferrington D. A., Kapphahn R. J., Leary M. M. (2016). Increased retinal mtDNA damage in the *CFH* variant associated with age-related macular degeneration. *Experimental Eye Research*.

